# Dosimetry-based treatment planning for molecular radiotherapy: a summary of the 2017 report from the Internal Dosimetry Task Force

**DOI:** 10.1186/s40658-017-0194-3

**Published:** 2017-11-21

**Authors:** Caroline Stokke, Pablo Minguez Gabiña, Pavel Solný, Francesco Cicone, Mattias Sandström, Katarina Sjögreen Gleisner, Carlo Chiesa, Emiliano Spezi, Maria Paphiti, Mark Konijnenberg, Matt Aldridge, Jill Tipping, Michael Wissmeyer, Boudewijn Brans, Klaus Bacher, Carsten Kobe, Glenn Flux

**Affiliations:** 1 Department of Diagnostic Physics, Oslo University Hospital, Oslo, Norway; 2 Department of Medical Physics and Radiation Protection, Gurutzeta/Cruces University Hospital, Barakaldo, Spain; 3 Department of Dosimetry and Application of Ionizing Radiation, Czech Technical University in Prague, Prague, Czech Republic; 4 Nuclear Medicine, Sant’Andrea Hospital, Department of Surgical and Medical Sciences and Translational Medicine, Sapienza University of Rome, Rome, Italy; 5 Section of Nuclear Medicine and PET, Department of Surgical Sciences, Uppsala University, Uppsala, Sweden; 6 Department of Medical Radiation Physics, Clinical Sciences Lund, Lund University, Lund, Sweden; 7 Nuclear Medicine Division, Foundation IRCCS istituto nazionale Tumori, Milan, Italy; 8 School of Engineering, Cardiff University, Cardiff, UK; 9 Department of Medical Physics, Pammakaristos Hospital, Athens, Greece; 10 Department of Radiology & Nuclear Medicine, Erasmus MC, Rotterdam, The Netherlands; 11 Nuclear Medicine/Radiotherapy Physics, UCL Institute of Nuclear Medicine and UCL Hospitals NHS Foundation Trust, London, UK; 12 The Christie NHS Foundation Trust, Nuclear Medicine, Manchester, UK; 13 Department of Nuclear Medicine, University Hospital of Geneva, Geneva, Switzerland; 14 Department of Nuclear Medicine and PET Center, University Hospital, Ghent, Belgium; 15 Department of Basic Medical Sciences, Division of Medical Physics, Ghent University, Ghent, Belgium; 16 Department for Nuclear Medicine, University Hospital of Cologne, Cologne, Germany; 17 Joint Department of Physics, Royal Marsden Hospital and Institute of Cancer Research, Sutton, UK

**Keywords:** Molecular radiotherapy, Treatment planning, Dosimetry

## Abstract

**Background:**

The European directive on basic safety standards (Council directive 2013/59 Euratom) mandates dosimetry-based treatment planning for radiopharmaceutical therapies. The directive comes into operation February 2018, and the aim of a report produced by the Internal Dosimetry Task Force of the European Association of Nuclear Medicine is to address this aspect of the directive. A summary of the report is presented.

**Results:**

A brief review of five of the most common therapy procedures is included in the current text, focused on the potential to perform patient-specific dosimetry. In the full report, 11 different therapeutic procedures are included, allowing additional considerations of effectiveness, references to specific literature on quantitative imaging and dosimetry, and existing evidence for absorbed dose-effect correlations for each treatment. Individualized treatment planning with tracer diagnostics and verification of the absorbed doses delivered following therapy is found to be scientifically feasible for almost all procedures investigated, using quantitative imaging and/or external monitoring. Translation of this directive into clinical practice will have significant implications for resource requirements.

**Conclusions:**

Molecular radiotherapy is undergoing a significant expansion, and the groundwork for dosimetry-based treatment planning is already in place. The mandated individualization is likely to improve the effectiveness of the treatments, although must be adequately resourced.

## Background

Radiopharmaceuticals have been used for the treatment of various forms of cancer and benign diseases since the 1940s [1, 2]. The level of radioactivity administered is primarily fixed, sometimes adjusted by body weight, body surface area, or clinical factors. Prescription levels for different treatments are commonly determined empirically, using similar approaches as for chemotherapy. The term “molecular radiotherapy” (MRT) has gained acceptance in recent years to describe the use of radiotherapeutics. Uniquely, for MRT, the patient-specific biodistribution can be determined by in vivo nuclear medicine imaging of the radiopharmaceutical, and basic biokinetics can also be monitored by external probes. Quantitative imaging of a tracer amount of either the radiopharmaceutical or of a companion diagnostic prior to therapy can enable the therapeutic administration to be chosen to achieve prescribed absorbed doses to different tissues. Post-therapy imaging enables the prescribed absorbed dose to be verified. Recently, the panel of mechanisms and targets for radiopharmaceuticals has increased significantly. Together with newly available radiotherapeutics, this has raised greater awareness of the field of molecular radiotherapy and in combination with technical developments has also rekindled the interest for patient-specific dosimetry in research settings and in clinical practice.

The European Council directive 2013/59/ Euratom mandates the use of dosimetry for treatment planning and verification [3]. In Chapter VII, Medical Exposures, Article 56, it is stated that:

“For all medical exposure of patients for radiotherapeutic purposes, exposures of target volumes shall be individually planned and their delivery appropriately verified taking into account that doses to non-target volumes and tissues shall be as low as reasonably achievable and consistent with the intended radiotherapeutic purpose of the exposure.”

Furthermore, from Chapter II, Definitions, Article 4, Definition 81, it is stated that:

“‘radiotherapeutic’ means pertaining to radiotherapy, including nuclear medicine for therapeutic purposes”.

The directive is due to come into force February 2018 and has been subject to considerable debate regarding interpretation and how the requirements may be fulfilled. Therefore, in 2015, the multidisciplinary Internal Dosimetry Task Force (IDTF) was established by the European Association of Nuclear Medicine (EANM) to address aspects of the 2013/59/ Euratom directive specifically concerned with dosimetry for MRT. The EANM IDTF consists of 17 members from Belgium, Czech Republic, Germany, Greece, Italy, The Netherlands, Norway, Spain, Sweden, Switzerland, Turkey, and the UK. Treatment-specific sections were drafted for 11 different categories of therapy procedures and indications (Table 1). The aim of the group was to identify the potential for patient-specific treatment planning and verification. While selected possibilities for imaging or other measurements and dosimetric methodology are included, no reviews regarding the optimal methods or the accuracy of the absorbed dose-effect relationships were performed. The specific pre- and post-therapy sections are therefore to be considered as suggested solutions and not definite guidelines. The report on the potential and prospects for dosimetry-based treatment planning for MRT will be made available on the EANM website. The aims of this article are to summarize the IDTF report and briefly review examples of treatment-specific dosimetric planning procedures.

**Table 1:** Molecular radiotherapies and possibilities for dosimetric treatment planning identified in the report

Radiopharmaceutical	Procedure	Compounds used for pre-treatment imaging	Post-treatment imaging or measurement methods
^131^I NaI	Benign thyroid disease	^131^I NaI ^124^I NaI ^123^I NaI	Thyroid probe. Gamma camera^a^ or SPECT/CT.
^131^I NaI	Differentiated thyroid cancer (DTC) with ablative intent and in the case of recurrent disease	^131^I NaI ^124^I NaI ^123^I NaI	Gamma camera or SPECT/CT. Whole-body probes, blood sampling
^131^I mIBG	Neuroblastoma in children and young adults	^131^I mIBG ^124^I mIBG ^123^I mIBG	Whole-body probe for bone marrow estimation. Gamma camera or SPECT/CT
^131^I mIBG	Neuroendocrine tumors in adults	^131^I mIBG ^124^I mIBG ^123^I mIBG	Gamma camera or SPECT/CT
^177^Lu DOTATATE	Neuroendocrine tumors	Radiolabelled somatostatin analogs	Gamma camera or SPECT/CT
^90^Y somatostatin analogs	Adult neuroendocrine disease	Analogs, as ^86^Y–DOTATOC ^111^In-DOTATATE	Bremsstrahlung gamma camera or SPECT/CT. PET/CT.
^89^SrCl_2_, ^153^Sm-EDTMP, ^186^Re-HEDP, and ^188^Re-HEDP	Bone pain palliation	^99m^Tc-MDP for ^153^Sm-EDTMP	Gamma camera or SPECT/CT for ^153^Sm-EDTMP, ^186^Re-HEDP and ^188^Re-HEDP
^223^Ra dichloride	Bone metastases from castration-resistant prostate cancer	^99m^Tc-MDP	Planar gamma camera imaging
^177^Lu PSMA ligands	Metastatic castration-resistant prostate cancer	Analog PET ligands	SPECT/CT
^90^Y microspheres	Liver metastases or primary tumors	^99m^Tc-albumin macro aggregate	Bremsstrahlung SPECT/CT. PET/CT.
^90^Y-ibritumomab tiuxetan	Non-Hodgkin’s lymphoma	^111^In-ibritumomab tiuxetan	Bremsstrahlung gamma camera or SPECT/CT. PET/CT.
^90^Y, ^32^P, and ^186^Re colloid, ^169^Er citrate	Radiosynovectomy	^99m^Tc MDP/HDP/HEDP and/or ^99m^Tc-HIG	Planar Gamma camera imaging, # dicentric chromosomes

^a^Gamma camera indicates both planar scintigraphy imaging and SPECT/CT imaging, if not otherwise specified

## Summary of report

The radiopharmaceutical procedures covered in the full report are listed in Table 1, which also displays selected post-therapy imaging and measurement methods that can form the basis for verification of absorbed doses delivered. Furthermore, the possibility for administration of a tracer amount of the radiopharmaceutical itself or of a “companion diagnostic” to estimate absorbed doses “up front” are indicated. Current clinical therapy regimens vary from single administrations to multiple cycles, and prescriptions range from the delivery of a fixed amount of radioactivity to dosimetric treatment planning. The range of current practice is reported in an IDTF survey on implementation of dosimetry procedures in Europe [4].

In the following sections, the potential for dosimetric treatment planning are summarized for five of the most common individual therapy procedures. In the full report, a brief introduction to the disease and radiopharmaceutical is given for each procedure, followed by a brief review of the reported effectiveness of the treatment, the potential for quantitative imaging that underpins normal tissue and target dosimetry, and existing evidence for absorbed dose-effect correlations. The potential for personalized dosimetry-based treatment planning is then considered. Finally, issues specific to the treatment are considered along with questions that merit further investigation. For the individual procedures included in the present summary, these have been condensed in two paragraphs: the first presenting the therapy and current practice, followed by a second describing the dosimetric procedures and dose-effects. Resource requirements are reported in a separate section.

### ^131^I NaI for the treatment of differentiated thyroid cancer with ablative intent and in the case of recurrent disease

The amount of ^131^I NaI activity to administer for differentiated thyroid cancer (DTC) treatment is commonly empirically determined. Typically, a fixed activity of ^131^I NaI ranging between 1.11 and 7.4 GBq is given [5]. In the case of recurrent disease, the administered activity may be calculated based on the absorbed dose constraints of the normal tissue, usually red marrow as recommended in EANM guidelines [6].

Radioiodine uptake in thyroid remnants and metastases can be determined with gamma camera planar imaging or with SPECT/CT which can provide more accurate quantification [7]. Camera calibrations and radiation protection measures may be demanded due to a high photon flux [7]. PET/CT imaging can also be performed using ^124^I NaI, which may be advantageous for determination of remnant mass and small metastases [8]. Currently, in the treatment of DTC, there are no well-established values for absorbed doses to remnants and metastases which may be used as prescription values. EANM guidelines recommend the calculation of red marrow absorbed doses from whole-body and blood dosimetry [6], and a constraint of 2 Gy is considered when using this methodology [9]. Additional organs at risk may include the lungs and salivary glands [10–12]. Issues regarding the alteration of biodistribution can occur due to the debated “stunning effect” for ^131^I NaI [13, 14] or due to prior administration of recombinant human thyroid-stimulating hormone (rhTSH) [15], which may have to be considered if treatment planning is performed.

### ^131^I mIBG for the treatment of neuroblastoma in children and young adults

The metaiodobenzylguanidine (mIBG) molecule structurally resembles norepinephrine (also called noradrenaline), and tumors expressing the norepinephrine transporter show mIBG uptake capacity. The prescription of ^131^I mIBG for neuroblastoma is often made according to whole-body absorbed dose, which is related to bone marrow absorbed dose and neutropenia [16].

If stem cell rescue is not scheduled, the main organ at risk is usually the bone marrow, with an absorbed dose constraint of 2 Gy [17]. An increasingly common protocol is to deliver a whole-body absorbed dose of 4 Gy in two administrations of activity separated by 2 weeks, followed by stem cell rescue. The first administration is delivered according to a body mass-based prescription of 444 MBq/kg [18]. The whole-body absorbed dose can be estimated by dose-rate measurements obtained with a probe. However, if there is bone marrow involvement, imaging is necessary to perform dosimetry. Quantitative imaging can be performed essentially as for ^131^I NaI, with the same technical considerations [7].

### ^177^Lu-DOTATATE for the treatment of neuroendocrine tumors

^177^Lu-DOTATATE is a radiolabelled somatostatin analog developed for the treatment of patients with somatostatin receptor-positive neuroendocrine tumors. The most frequent treatment protocol is currently to administer 7.4 GBq for up to four times with a 6 to 10-week interval between each administration [19]. However, protocols delivering cycles of 7.4 GBq until a maximum prescribed absorbed dose to the kidneys and the bone marrow is reached are under investigation [20].

Although the photon yield is relatively low, the high level of activity administered makes quantitative gamma camera imaging of ^177^Lu possible [21, 22]. Late treatment-related kidney toxicity has not been reported for patients receiving a kidney absorbed dose over 28 Gy, a commonly used tolerance limit, indicating that this may be a conservative value [23]. A clear correlation between tumor absorbed doses and the response to the treatment was reported in pancreatic neuroendocrine tumors [24]. Although it has been demonstrated that patient-specific absorbed doses for ^177^Lu can be calculated and have a clinical benefit, the absorbed dose limits for normal tissue and the desirable absorbed dose to the tumors require further investigations.

### ^223^Ra dichloride for the treatment of bone metastases from castration-resistant prostate cancer

^223^Ra dichloride is an alpha-emitting radiopharmaceutical approved for the treatment of patients with castration-resistant prostate cancer, symptomatic bone metastases, and no known visceral metastatic disease. Being an analog to calcium, cationic radium is taken up by areas of increased osteoblastic activity. A fractionated approach is routinely used for the delivery of this treatment with six administrations of 55 kBq/kg body weight.

The low yield of photons, combined with the low amount of activity administered, makes quantitative imaging of ^223^Ra challenging. However, it has been demonstrated that prolonged gamma camera imaging is feasible [25, 26] and that activity can be quantified to within 20–50%, depending on the volume imaged. A study showed that absorbed doses delivered to normal organs vary by an order of magnitude between individual patients [27]. Uptake of ^223^RaCl_2_ in metastases has been seen to correlate with that of ^99m^Tc MDP [28], demonstrating a potential for treatment planning. There is no evidence as yet of correlations between the absorbed doses delivered and effect. Besides developing reliable dosimetric methodology for this treatment, the relative biological effect is yet to be determined, and the short range of the alpha emissions necessitates investigations of microdosimetry and small-scale dosimetry.

### ^90^Y microspheres for the treatment of primary and metastatic liver cancer

Intra-arterial locoregional liver therapies have their rationale in the fact that liver lesions are fed mainly by the arterial stream, while normal parenchyma is supplied by portal vein blood flow. At present, both ^90^Y glass microspheres and ^90^Y resin microspheres are used, both licensed as medical devices to treat liver primary hepatocarcinoma (HCC) and liver metastases [29]. Toxicity is of particular importance, as patient death from radioembolization-induced liver disease leading to liver failure can be a consequence of a standard treatment [30]. With current recommendations, dosimetry is sometimes used as a basis for treatment planning, although methods for absorbed dose prescription vary.

Simulation scanning is performed with ^99m^Tc-albumin macro aggregate (MAA) administered under angiographic guidance for quantitative imaging and pre-treatment dosimetry [29]. The permanent trapping into liver capillaries of the ^99m^Tc MAA and of the therapeutic ^90^Y microspheres allows dosimetry to be performed from only one scan [30]. Post-therapy quantitative imaging can be performed by ^90^Y bremsstrahlung SPECT or ^90^Y PET with suitable corrections [31, 32]. Correlations between the absorbed doses delivered and toxicity and response have been reported both for hepatocellular carcinoma [33–35] and colorectal metastases [36, 37].

### Resource requirements

Implementation of dosimetry for therapy, particularly on a routine basis, has implications for infrastructure resourcing. The level of resources required will depend on the complexity of the dosimetry procedure and will vary according to local and national protocols and guidelines. Each procedure will have resource implications for both an initial setup of a dosimetry service and for ongoing support.

Resources fall into categories of equipment and staff. Whole-body dosimetry may be performed with a portable or externally mounted compensated Geiger counter which enables multiple measurements to be made by either staff or, if necessary, possibly by comforters and carers. Both the legal implications and the technical complexity should be carefully considered if the latter group is to perform such measurements. Blood dosimetry may be derived from samples measured in a well counter. Image-based dosimetry requires a gamma camera or PET system that has been set up for the radionuclide under investigation at activity levels relevant to the procedure. In addition to routine quality control procedures, this entails determination of calibration factors for image quantification and characterization of camera deadtime which is important for post-therapy imaging. For each therapeutic procedure, the required number of measurement time points (especially imaging time points) needs careful investigations as this directly affects both the dosimetric accuracy and the resource implications. Unlike many of the other resource requirements, this also impacts the time spent by the patients. Volume estimation may be acquired from radiological images or, in the case of radioiodine treatment of benign thyroid disease, from ultrasound scanning as well as from the SPECT and PET data.

As a multidisciplinary area, a range of trained staff are necessary to provide a comprehensive service. These include medical physicists for image quantification and absorbed dose calculations, nuclear medicine technologists and radiographers with experience in quantitative imaging, nuclear medicine physicians and radiologists for performing or supervising volume outlining, and possibly other specialists to contribute on patient-specific prescriptions and procedures.

## Discussion

The potential for dosimetry-based treatment planning was demonstrated for all therapy procedures, using either the same radiopharmaceutical or diagnostic analogs. Both planning and verification can be technically demanding due to various factors, including low activity levels in patients and/or low photon yield, image co-registration, calibration of the system, volume definition, choice of imaging time points, and kinetic modelling. However, extensive work has laid the foundation for individualized dosimetry of MRTs.

Absorbed dose-effect relationships have been determined for many therapy procedures in single-center studies, and multicenter clinical trials are necessary to gather further evidence and substantiate these findings. However, relevant data can be collected by simply performing post-therapy imaging and dosimetry routinely in current practice and by comparison of the absorbed doses delivered with response and toxicity data. For many MRTs, a requirement for post-therapy verification may therefore represent a rational and feasible manner of initiating a dosimetry-based treatment planning program.

In conclusion, molecular radiotherapy is undergoing a significant expansion. Many new radiotherapeutics are being introduced into the clinic and an increasing number of patients are being treated for common as well as rare cancers. This will have a significant effect on healthcare funding, patient management, and the logistical and scientific challenges faced by nuclear medicine departments and their collaborators. As medicine in general has begun to focus on personalized treatment, often accompanied by molecular imaging, this growth in radiotherapeutics offers unprecedented opportunities for recognition, support, and significant development. Nuclear medicine combines diagnostics and therapeutics and provides unique possibilities for personalized treatment. The EANM IDTF report provides an overview of the potential and prospects for dosimetry-based treatment planning of MRT, revealing that the groundwork is already in place. The report focuses on the overall aspects of treatment planning, and further work may be required to complement the status summary. Topics of interest include the use of companion diagnostics, resource implications, the study of absorbed dose-effect correlations, refinement of technical aspects including image quantification and voxel-based dosimetry, biophysical considerations including biologically effective dose (BED) calculations, education, and training. These investigations are important to ensure the clinical and cost-effectiveness of new radiotherapeutics, as well as for established therapy procedures. However, MRT has the unique potential to provide patient-specific molecular information allowing guidance for personalized treatment.
